# Uneven stigma loads: Community interpretations of public health policies, ‘evidence’ and inequities in shaping Covid-19 stigma in Vietnam

**DOI:** 10.1016/j.ssmph.2022.101270

**Published:** 2022-10-13

**Authors:** Duy Hoang Trinh, Shannon McKinn, Anh Thuy Nguyen, Greg J. Fox, Anh Thu Nguyen, Sarah Bernays

**Affiliations:** a Woolcock Institute of Medical Research, Ha Noi, Viet Nam; b Sydney School of Public Health, Faculty of Medicine and Health, University of Sydney, Sydney, Australia; c Central Clinical School, Faculty of Medicine and Health, University of Sydney, Sydney, Australia; d Department of Global Health and Development, London School of Hygiene and Tropical Medicine, London, UK

**Keywords:** COVID-19, Stigma, Public health policies, Qualitative, Inequity, Vietnam

## Abstract

The infectious spread of COVID-19 has been accompanied by stigma in both global and local contexts, sparking concern about its negative effect on individuals, communities, and public health responses. The changing epidemiological context of the COVID-19 epidemic and evolving public health responses during the first year of the pandemic (2020) in Vietnam serve as a case study to qualitatively explore the fluidity of stigma.

We conducted in-depth interviews with 38 individuals, (13 cases, 9 close contacts, and 16 community members) from areas affected by local outbreaks. Thematic analysis was conducted iteratively.

Our analysis indicates that the extent and impacts of COVID-19-related stigma were uneven. Adapting the clinical term 'viral load' as a metaphor, we describe this variation through the wide range of 'stigma load' noted in participants' experiences. Individuals encountering more acute stigma, i.e. the highest 'stigma load', were those associated with COVID-19 at the start of the local outbreaks. These intensively negative social responses were driven by a social meaning-making process that misappropriated an inaccurate understanding of epidemiological logic. Specifically, contact tracing was presumed within the public consciousness to indicate linear blame, with individuals falsely considered to have engaged in 'transgressive mobility', with onward transmission perceived as being intentional. In contrast, as case numbers grew within an outbreak the imagined linearity of the infection chain was disrupted and lower levels of stigma were experienced, with COVID-19 transmission and association reframed as reflecting an environmental rather than behavioural risk.

Our findings demonstrate the role of public health policies in unintentionally creating conditions for stigma to flourish. However, this is fluid. The social perceptions of infection risk shifted from being individualised to environmental, suggesting that stigma can be modified and mitigated through attending to the productive social lives of public health approaches and policies.

## Highlights

• Those associated with COVID-19 experienced variable degrees of stigma - 'stigma load'.
• Those linked to COVID-19 at the start of outbreaks experienced highest stigma load.
• Misinterpretation of public health strategies contributed to blaming discourses.
• Stigma receded when risk framed as environmental not behavioural.
• Fluidity of stigma suggests potential to allay stigmatising effect of interventions.

## 1 Introduction

The unfolding of the COVID-19 pandemic was accompanied in many places by the spread of related stigma in global and local contexts (Bagcchi, 2020; Peprah & Gyasi, 2020; Roelen et al., 2020; Villa et al., 2020). Although the impacts of COVID-19 prevention and infection were intensely social as well as clinical, early public health strategies often, and short-sightedly, marginalised insights from social science (Pickersgill et al., 2022). In order to learn lessons from the early responses in the COVID-19 pandemic to strengthen future pandemic preparedness, it is critical to take a social science lens to examine how public health strategies may have unwittingly supported the development of stigma, causing further social damage to those affected but also risking undermining the effectiveness of its strategies.

The epidemiological trajectory of COVID-19 in Vietnam in 2020, which was characterised by discrete outbreaks, makes it an exceptional case study to explore the interaction between public health policies and the fluid constructions of social meanings about those associated with COVID-19.

### 1.1 COVID-19 stigma: what we know and are yet to know

Goffman's original conceptualisation of stigma as the "spoiling of identity" is commonly understood as the relational discreditation and devaluation of an individual's social identity resulting from their possession of an attribute or group membership (Goffman, 1963). Stigma is often typified by the aligning of difference with the perceived moral inferiority of the individual, which reflects the local moral context and landscape. It is the framing of this attribute as inferior or undesirable that enables ostracization or shunning of ‘identified’ individuals, inferring a loss of their social position that can be either temporary or have more enduring social consequences. It is manifested in *enacted stigma*, which is overt discrimination and subtle social devaluation such as being avoided, patronized or treated unkindly, and *felt stigma*, which is people's awareness and expectation of enacted stigma from others accompanying feelings of anxiety and fear (Quinn & Earnshaw, 2013; Scambler, 2009).

The earliest signs of COVID-19 stigma, in which an individual is socially discredited through their association with COVID-19 infection, was evident in racism and discrimination against the Asian community as perceived ‘origin groups’ (Budhwani & Sun, 2020; Choi, 2021). There has been increasing evidence documenting stigmatization targeted at particular social groups, intersecting with existing identities (Turan et al., 2019). It has affected a wide range of groups, including but not limited to survivors of infection (Abdelhafiz & Alorabi, 2020; Bhanot et al., 2021; Jesus et al., 2021), healthcare workers (Dye et al., 2020; D. McKay & Asmundson, 2020), as well as those defined by their gender (Edmond, 2021; Solomon et al., 2021), and region (Fan et al., 2021). Although the intersectionality lens is often used to explain the complex social processes underpinning existing stigma, less attention has been applied to illuminating the mechanisms through which stigma related to new diseases emerge to intersect with social identities.

That COVID-19 stigma would emerge as a variable characteristic of the pandemic was predicted by many. Recognising that attempts to address misinformation and mitigate flourishing stigma through educational campaigns tend to have a modest and transient impact in changing attitudes (Gronholm et al., 2017), there were early calls to act pre-emptively to disrupt the emergence of stigma from the start through designing public health strategies and policies to avoid and correct assertions of blame directed at groups associated with emerging infections (Hargreaves et al., 2020; Huda et al., 2020; Roelen et al., 2020; Villa et al., 2020).

This included warnings against using terms in the COVID-19 response such as “patient zero,” and “super spreader”, which had previously been shown to have toxic social effects (Carinci, 2020; R. McKay, 2020). There were also criticisms that adopting the tuberculosis identification strategy as part of the COVID-19 response could arguably legitimize a “witch-hunt”-like hysteria through transferring stigmatising language from tuberculosis (TB) control (Sotgiu & Dobler, 2020). Although concerns were raised, it rarely changed the early implementation of public health strategies and the growing literature on COVID-19 stigma in the first years of the pandemic (Bhanot et al., 2021) indicates that there was a failure to effectively respond to the warnings that there were social consequences to public health framings. Justified by the urgency of the situation, the hard-won lessons from scholarship and advocacy on HIV stigma that the language that circulates within clinical and social descriptions of infectious diseases within lay discourses are not neutral and can undermine engagement and the effectiveness of public health strategy (Bernays et al., 2021; Wakeman, 2019), were not given due credence.

As the pandemic played out, different public health strategies have been employed, and became a primary instrument through which society navigated uncertainties. There is a need to understand the role of public health policies as a potential moderator in the emergence of stigma rather than being removed from the production of stigma (Logie & Turan, 2020; Tomczyk et al., 2020). Public health policies, like public health language, are not socially neutral but inevitably have a social impact. Exploring the relatively novel emergence of COVID-19-related stigma and examining the policy context in which it arises presents an opportunity to better understand the pathogenesis of stigma, a longstanding blindspot (Keusch, Wilentz, & Kleinman, 2006). This is likely to develop our conceptualisation of the dynamism of emerging and altering stigma in relation to various and novel health conditions (Farrimond, 2021; Holzemer et al., 2007; Whitehead et al., 2001).

Adopting Farimond's argument (2021) that the content and the severity of stigma can be amplified or mitigated depending on structural forces (e.g. social prejudices, anti-stigma intervention, public health policies), we consider what can be learned from identifying the drivers of variation and strength in shaping individuals' experiences of COVID-19 related stigma. While stigma operates relationally, it has been primarily studied via viewpoints of the stigmatized rather than the stigmatiser. In studying how stigma arose and was experienced in real-time from the perspectives of both those associated with COVID-19 (stigmatized) and those who were able to distance themselves from COVID-19 associations (who were often themselves stigmatisers), we focus on the factors which supported stigmatising attitudes as they circulated, and changed, within the community.

### 1.2 Stigma and the changing epidemic in Vietnam

To contextualise our study, we first outline the broad characteristics of the population in Vietnam, before describing the key features of the 2020 COVID-19 pandemic in Vietnam.

Vietnam, a country with an informal economy estimated at around 20%, has a population of 97 million people of which a third of the population is employed in the agriculture sector. The literacy rate was 95.4 in 2020. Despite there being 54 ethnic groups within the Vietnamese population, the predominant group are the Kinh people who make up 85% of the population and reside primarily in the urban areas. Vietnam is a socialist republic and classified as an upper-middle-income country.

Economic reform in 1986 is credited with improving the general health of the population. Over the subsequent decades, public healthcare has become increasingly fragile with the growing reliance on a system of user fees, which is considered to have contributed to intensifying health inequality between groups (Van Minh & Nguyen-Viet, 2017). However, Vietnam was lauded as a national success story for the effective deployment of public health strategies to contain the disease within the first year of the pandemic (Nguyen Thi Yen et al., 2021; Tran, Le, & Nguyen, 2020).

**Fig. 1:**
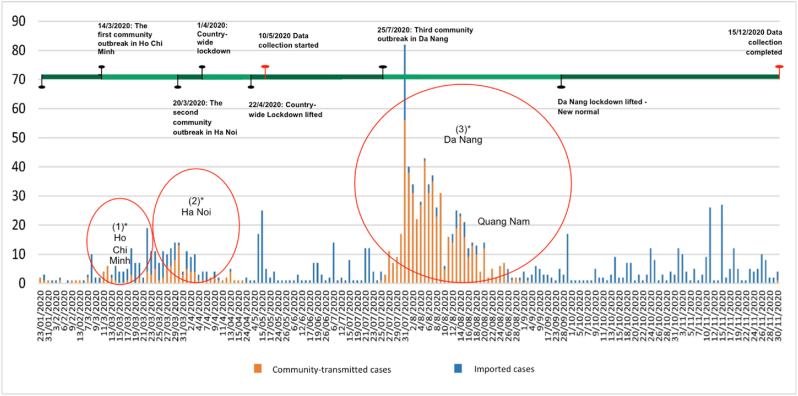
Three COVID-19 waves in Vietnam for the year 2020 and timeline of public health responses (adopted from T. A. Nguyen, Tran, et al., 2021) *indicates 1st, 2nd and 3rd outbreaks.

Vietnam’s COVID-19 epidemiological trajectory in the first year provides an exceptional case study through which to examine the factors shaping the emergence and dynamism of stigma. In 2020, Vietnam experienced three waves (outbreaks) of COVID-19 infection (i.e. with community spread), which progressively increased in scale (Fig. 1), in the three major cities of Ho Chi Minh, Ha Noi and Da Nang, situated respectively in the south, north and central regions of Vietnam. These cities are economic centres with high population densities, and vibrant tourist and industrial activities. The third and largest outbreak in Da Nang also spread to the rural neighbour province of Quang Nam. In 2020, COVID-19 infections were largely concentrated within the younger population as acquisition risk was connected to the relatively high mobility and denser social networks of younger people within these three outbreak locations (Van Nguyen, Tran, et al., 2021).

The public health policies implemented during the first year of the COVID-19 pandemic in Vietnam were diverse (Le et al., 2021; L. T. T. Tran et al., 2021), and shifted from targeted to more universalistic in response to the real-time situation. Swift containment strategies were deployed at the early stage of each outbreak (when there was still a relatively low caseload). This relied on the execution of timely and systematic contact tracing (T. A. Nguyen, Tran, et al., 2021).

Vietnam's contract tracing classification marks index cases as F0 and individuals with first-, second-, and third-generation COVID-19 exposure (close contacts) named as F1, F2, and F3 respectively (T. V. Nguyen, Tran, et al., 2021). Government mandated health declarations, in which people had to submit their medical information and travel history. Information about index cases was disseminated in the media to aid the self-identification of risk within the community so that individuals could report to health authorities and isolate. These policies were intensely focused on breaking the chains of infection, with limited overt consideration of the social consequences. A characteristic acknowledged by Vietnam's Ministry of Health, which has since conceded that these policies were socially insensitive (Vietnam Ministry of Health, 2021). Reflecting epidemiological principles that contact tracing becomes a less effective strategy as case numbers grow, alternative strategies such as localised lockdowns and mass community testing to identify cases were prioritised, and the provision of public information about individuals ceased, as outbreaks grew.

Public health prevention messaging was provided in the national language – Vietnamese – and so was accessible to the majority. This has been identified as contributing to the success of Vietnam's epidemic containment throughout 2020 (Tam et al., 2021). Some credited the Government's approach to stimulating social solidarity, directly evoking calls to the country's collectivist national identity (Small & Blanc, 2021). However, the effectiveness of these approaches may be overstated and risk over-simplifying the social response because the appeal to solidarity tends to obscure the significance of high-profile instances of circulating stigma within the early stages of the pandemic in Vietnam (Max, 2020). And it does not explains why there were parallel efforts in tackling misinformation to redress the rumours fuelling the stigmatization of affected individuals (T. H. D. Nguyen & Vu, 2020).

## 2 Methods

### 2.1 Study Setting

This qualitative study was carried out as part of the V-COVID Program of Research (Understanding COVID-19 in Vietnam) which aimed to inform the Vietnamese Government's response to the COVID-19 outbreak. The qualitative study began shortly after the end of the first national lockdown in early May 2020 and ran until the end of the year, in areas that had recorded community transmission in localised outbreaks. Such responsive and rapid research is needed to understand evolving situations, such as the needs of those affected by the pandemic (Richardson et al., 2021, p. 19).

### 2.2 Study design

A qualitative methodological approach, using individual in-depth interviews, was adopted to investigate the lived experiences of those affected by COVID-19 and the public health response. This included individuals who had confirmed positive diagnoses, close contacts, and community members living in outbreak locations. The study aimed to understand the social meanings that emerged around those associated with COVID-19, including the influences and drivers of COVID-19-related stigma.

### 2.3 Sampling and recruitment

This study overall adopted purposeful sampling strategies. In order to reflect the variation in the situational contexts, different pragmatic sampling strategies were adopted (Patton, 2014). Initially, we adopted a convenience sampling approach to identify participants through existing social networks of colleagues at the Woolcock Institute in Ha Noi, Vietnam. Initial participants then acted as seeds to invite other close contacts and confirmed cases within their social networks to participate. During the latter half of the data collection period, the parent study began conducting a sero-surveillance study. Drawing on the demographic information collected within the parent study we adopted a purposive and theoretical sampling approach to recruit the remainder of our sample, based on our emerging areas of interest. We sought to identify individuals directly affected by COVID, either as cases or close contacts, through social networks of community members and local healthcare workers.

Given the sensitivity at the time of being publicly associated with COVID-19, we encountered a relatively high number of refusals and had to employ a convenience-based sampling approach, so that anyone fitting our criteria and willing to participate was interviewed. We approached recruitment with appropriate sensitivity to maintain the anonymity of those we approached as cases or close contacts. Unless a participant expressed a clear interest in participating, we did not revisit them to invite them again. Reflecting the primary characteristics of those infected, this group was predominately of working age (20–64) with relatively high mobility. We also deliberately recruited as diverse a group of community members, residing in outbreak locations, as feaisble to be able to explore the attitudes of those living there. This enabled us to recruit some older individuals to increase the breadth of experiences explored within this study.

## 3 Ethical approvals

Ethics approval was obtained in both Vietnam (Ministry of Health – National Hospital for Tropical Diseases 10/HDDD-NDTU) and Australia (University of Sydney, 202/354).

### 3.1 Data collection and analysis

A total of 38 interviews were conducted by two Vietnamese researchers (DHT and AN). In line with the public health restrictions in place during the data collection period, we conducted all interviews by telephone or voice call on Zalo (a domestic Vietnamese language social media platform). One participant withdrew consent and their data were immediately deleted. Participants were asked to find a space in which they felt comfortable speaking openly. Interviews lasted between 30 and 60 min and were audio-recorded.

Interviewers used a flexible topic guide which was iteratively revised based on systematic debriefing discussions (described below) and initial analyses. After each interview, detailed interview summaries based on audio recordings and interviewer field notes were written and shared across the research team for discussion. The research team held systematic debriefing meetings every two to three interviews to develop analytical ideas and refine the topic guide (see the supplementary document.) (McMahon & Winch, 2018).

**Fig. 2:**
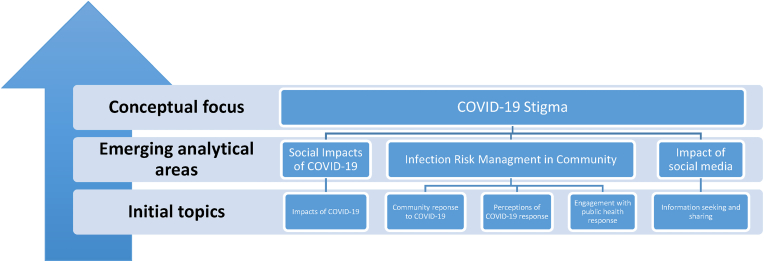
Development of analytical focus.

Besides informing ongoing data collection and identifying thematic saturation, this iterative data analysis allowed us to further refine our area of focus. Having begun our interviews with a broad focus to explore the experiences of being affected by COVID either directly through association with infection or indirectly through being a member of an affected community, we honed our focus to narrower topics through our iterative data collection and analysis approach. This is reflected in Fig. 2.

All interviews were transcribed and translated into English. The research team developed a coding framework that was then applied across the dataset by the first author. Coding was conducted using the Microsoft Word comment function. Coded data were systematically organized and charted using Google Sheets and discussed within further analysis meetings with the research team. Themes were developed through looking at relationships between codes attending to any alternative explanations and outliers, prior to being discussed, and revised by other authors.

## 4 Results

### 4.1 Sample

Reflecting the epidemiological trajectory of the first year of the pandemic in Vietnam, participants mainly came from areas affected by local COVID-19 outbreaks in Ha Noi and Da Nang (Table 1). Participants were categorised into three groups in relation to their association with COVID-19 including former confirmed cases (referred to as COVID-19 survivors hereinafter), former close contacts, and community members.

**Table 1:** Sample distribution by waves of infection and corresponding location.

Approximate Time of recruitment	2nd Outbreak		3rd Outbreak		TOTAL (%)
Location	Ha Noi	Ho Chi Minh	Da Nang	Quang Nam	
COVID-19 status					
Confirmed Case	3	0	8	2	**13 (34%)**
Close Contact	7	2	1	0	**10 (26%)**
Community member	11	0	4	0	**15 (40%)**
TOTAL (%)	**21 (55%)**	**2 (5%)**	**13 (35%)**	**2 (5%)**	**38 (100%)**

We interviewed 28 individuals, with a relatively even gender distribution (18 men, 20 women) across the sample. But a quarter of the men that we approached (6 out of 24) refused to participate, which was higher than the refusal rate of women (2 out of 22). This possibly reflects how the sensitivity of the topic was managed, a point we return to later. Although there was a relatively broad range of ages included (20–60), there was limited participation from elderly participants (65+). This was in part because they tended to only be recruited within the third category (community members), but it is feasible that their inclusion may have been hampered by a reliance on phone interviewing. Participants came from a wide range of socioeconomic and professional backgrounds including both formal and informal sectors accounting for 67% and 37% of the sample respectively.

### 4.2 Inverted correlation between COVID-19 ‘stigma load’ and epidemiological time

The survivors and former close contacts were interviewed approximately 3–4 weeks after their official medical confirmation that they were free from contagion risk (e.g., discharge from treatment/quarantine facilities). The overwhelming majority of COVID-19 survivors and former close contacts reported that they had experienced some negative reactions from others because of their association with the infection. However, the degree to which individuals reported having experienced being stigmatised was neither universal nor consistent.

**Fig. 3:**
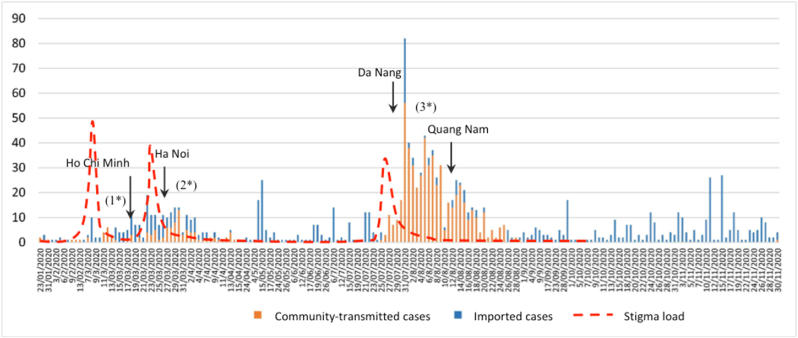
The stigma load against three waves of outbreaks (adopted from T. A. Nguyen, Tran, et al., 2021) *indicates 1st, 2nd and 3rd outbreaks.

The variation in stigma load followed a particular pattern, which was not defined by the status of COVID-19 risk acquisition but rather by the moment of identification within a local outbreak. We refer to this as a particular point in ‘epidemiological time’ to depict the importance of the moment within an outbreak or ‘wave’ of infections that were identified as being directly associated with COVID-19 infection. The earlier in the local outbreak one was identified, the more likely he/she reported experiencing severe stigmatising reaction: the later in the outbreak, the lower the intensity of stigma they encountered from others. To reflect this variation, we use the term *stigma load*. This is an intentional adaption of the clinical measurement of viral load, which we deploy as a metaphor to depict the dynamic nature of stigma related to infectious disease, shown by the inconsistency across cases, and over time within cases. Fig. 3 simulates approximately the pattern of stigma load when mapped onto former survivors and close contacts' reported experiences within epidemiological time.

In the next two sections (4.2.1 and 4.2.2), we describe participants' lived experiences to illustrate varying ‘stigma load’. In the final section, we explore why and how participants' experiences of stigma broadly correspond to specific public health strategies being pursued at the time of their identification.

#### 4.2.1 High ‘stigma load’ in cases occurring earlier in outbreaks

Experiences of acute stigma were concentrated among a minority group of participants who were identified as having been exposed to COVID-19 early in a localised outbreak or when case numbers of COVID-19 infection in the community were low (Table 2). These individuals had usually been detected when incidence was still relatively low either through contact tracing or mass testing surveillance, and their personal information was likely to be shared publicly via various media outlets. Although ostensibly anonymized, the provision of this information generated enormous public attention and commonly facilitated deductive disclosure of participants’ identities. During a period of potential clinical vulnerability, these individuals became socially exposed to intense public scrutiny of their behaviour. They often encountered avoidance, dismissal, name-calling, and verbal harassment by other people within their community.

*"People know that I had COVID-19. I heard that they were afraid that I would bring it to the village and infect them, so they stayed away from my family. No one wants to associate with me anymore … No one has called or talked to us ever since, even our relatives have been avoiding us."* (COVID-19 survivor identified early in 3^rd^ outbreak, female, 45, Quang Nam)

**Table 2:** Stigma load distribution by the epidemiological time of identification among affected individuals (total of 22).

Time of identification within an outbreak	Earlier (low caseload)		Later (high caseload)	
*COVID-19 Status*	Survivor	Close Contact	Survivor	Close Contact
*Stigma load reported*				
Higher	**5**	**2**	1	0
Lower	0	0	**7**	**7**

Much conjecture was invested in interpreting their motivations and the majority of these individuals were presumed to have deliberately infected others. They were subjected to vitriolic discourses on social media and accused of being personally responsible, through their perceived “excessive mobility”. Accused of “bringing the virus to the community” they were blamed for the subsequent spread of the virus within an outbreak.

*"People here are terribly stigmatized. They said a lot of cruel things about patient 243 [the first patient identified by the local health authority in a community outbreak in Ha Noi]. They said he brought the disease to the village."* (Close contact identified later in 2^nd^ outbreak, 50, female, Ha Noi)

For those who developed only a mild infection or for close contacts who remained negative, their avoidance of severe infection did not necessarily protect them from substantial social harm. Nor, for those infected, did their ‘stigma load’ necessarily recede with physical recovery. Despite being cleared of the infection, some participants continued to be perceived as an ongoing risk. This led to their continued exclusion from social and work activities. For many survivors and former close contacts, the implications of association lingered like ‘long social COVID’.

*“It [being a close contact] gives me a bad reputation. It has a spill-over effect on other aspects of my life. Now, despite no lockdown or social distancing, everyone keeps telling me not to do things. They told me ‘stay home, don’t go to your dance studio, who knows what you may do again.’ I can feel prejudice against me. They often make half-joke statements about me being ‘naughty’ because they think I may do something frivolous again that may cause troubles.”* (Close contact identified early in 2^nd^ outbreak, 24, female, Ha Noi)

There appeared to be a gendered dimension to participants' reported experience of COVID-19-related stigma. A strong pattern within our dataset was that female participants reported higher levels of distress concerning the potential social damage of their association with COVID-19. Among the sample, the reported degree of exposure to potentially stigmatising experiences appeared to be relatively similar between male and female participants, shaped by the point of their identification within a local outbreak rather than their gender. Rather it may reflect that there was a gendered response to potential stigmatising experiences. This does not indicate that enacted stigma disproportionately affected women. While female participants openly admitted their anxiety that being associated with COVID-19-would tarnish their reputations, male participants tended to minimise the effect of their reported stigmatising experiences and repeatedly dismissed being concerned about social judgment, as exemplified in the following quote from a male close contact:

*“When I touched a random wall, they [the neighbours]) sprayed [to disinfect] it immediately. They even blocked the windows of my house [without permission]. It was exaggerated … But I didn’t care or pay attention to what they said … but I guess I feel a little bit guilty [for being a close contact].”* (Close contact identified early in 2^nd^ outbreak, male, 35, Ha Noi)

This pattern may in part explain the higher rates of refusals observed among eligible men. Brief discussions with male refusers suggested that they downplayed the relevance of their own experiences, citing the lack of social impact it had had on their lives. This may have been shaped by gendered social norms about how males and females *should* present themselves, which reflect cultural values in Vietnamese society. It may also reflect the amount of gendered social capital that they can draw on to deflect stigmatising discourses, which warrants further attention.

#### 4.2.2 Decreasing ‘stigma load’ across the epidemiological time of an outbreak

In our sample, the majority of COVID-19 survivors and close contacts who had been identified within the later stages of a localised outbreak, where incidence was higher, described experiencing comparatively more tolerable social responses. As the number of cases grew within an outbreak the public health strategy shifted away from contact tracing. Although this did not mean the absence of stigma, it diffused the vitriol directed at those affected with attention becoming less overtly negative, and more localised and ambivalent.

*“Only my family and my friends recognized me, they took pictures of the news reporting my information and sent it to me. There were my initials and location on it … But I don't see it [publishing personal information] as a problem. There were no rumours about me.”* (COVID-19 survivor identified early in 3^rd^ outbreak, male, 28, Da Nang)

*“To be honest, as I have become a patient myself, I understand the feeling of sick people like Mr. [name - patient 243]. When I was not sick, he was discriminated against by people in the village. Only for patients like me who tested positive following him, people don’t say much about us. But now people still say that he is the one bringing the disease to our village.”* (COVID-19 survivor identified later in 2^nd^ outbreak, 58, female, Ha Noi)

Once participants who were identified later in a particular outbreak had physically recovered and were released from quarantine, the majority experienced relatively little stigma. They reported encountering limited social dislocation upon their return, and many described being welcomed back by their community. Encountering negative responses was the exception rather than the norm.

However, having observed the vitriol directed at those identified early within an outbreak, felt stigma, for some, remained high. To mitigate this, a small minority in this group, prolonged their isolation beyond the current public health requirements to distance themselves from potential accusations.

*“When I came back from the quarantine camp [as an overseas returnee], I quarantined myself for another three weeks instead of two weeks just in case. The health worker at my local health centre told me that there was no mandatory home quarantine in my case. But I did it anyway. I am afraid that people might see and judge me for going out ‘too soon.’”***(**Close contact identified later in 1^st^ outbreak, 27, female, Ho Chi Minh)

### 4.3 Explaining variation in ‘stigma load’: social sense-making of public health strategies

The variation observed in participants' experiences of stigma correlated with different epidemiological contexts. We argue that the public health strategies and messaging, although presented ostensibly as neutral, became imbued with social meanings as the ‘public’ projected powerful interpretations onto these strategies. We demonstrate this by explaining how *linear blame* (4.3.1) and *projected intent* (4.3.2) arose from an inaccurate interpretation of public health messages about contact tracing. These misinterpretations were amplified within the media (3.3.3), causing substantial social harm for those identified early in an outbreak. As public health strategies adjusted in line with epidemiological trends over the course of an outbreak, these interpretations shifted towards framing acquisition as an environmental risk (3.3.4).

#### 4.3.1 Linear blame: the social amplification of ‘stigma load’ through contact tracing

Participants' accounts of experiencing high ‘stigma loads’ corresponded with being identified through the public health strategy of contact tracing. The earliest identified cases within an outbreak in a community were often scientifically referred to as “index cases”, an epidemiological term indicating an individual first noticed by the health authorities within a certain cluster. Yet these individuals were widely assumed to have been the ones who “brought the virus into our community,” which in epidemiological terms would be a “primary case.” This confusion among the public in their understanding of terms concerning the ‘start’ or source of the infection in a community shaped assumptions about perceived culpability.

*"People also wonder a lot about how the virus was transmitted. He may be the first carrier of the disease because he is the first patient in the village, the starting point. Because he is the F0 [index case] of the village, they said he brought the disease to our village."* (COVID-19 survivor identified later in 2^nd^ outbreak, female, 37, Ha Noi)

Participants' accounts reflected the misappropriation of widely publicised epidemiological information accompanying the process of contact tracing. It was co-opted as a mechanism to ‘make sense’ of who was to blame, imposing a social order that inflicted a severe penalty on early-identified individuals. The acute focus on personally identifying individuals, distorted by rumours and accusations, was justified by the perceived position as a threat to national security, further exacerbating the social harms of association.

*“When I returned from the epicentre of Da Nang, people in my area searched everywhere for F1 [close contact] like me, like we are criminals."* (Close contact identified early in 2nd outbreak, female, 39, Ha Noi)

#### 4.3.2 Projected individual intent: assumptions of transgressive behaviour

The blame towards individuals who were identified early in the outbreak was underpinned by a strong social conviction that they must have acted irresponsibly to acquire and transmit it. What constituted 'responsible behaviour' was re-framed and adjusted over the course of the first year of the pandemic. The emergence of dynamic behavioural criteria against which the 'reported' actions of individuals were evaluated was formed in response to the evolving national guidelines on movement restrictions and hygiene practices. It was enabled, in part, by the incomplete evidence-base. Accusations of intentional 'risk' tended to be divorced from their explanatory context and did not attend to the asymptomatic nature of many infections. Crucially they were also co-opted to align with and selectively reinforce existing social prejudices.

*"We were shocked. It was 99 days without community transmission, how could another (the third) outbreak happen like that! We were even more shocked by the news that Da Nang ‘could not find the first F0 [index case]’ We didn't understand why. But we kept thinking about the illegal Chinese immigrant, they must be the ones who caused this outbreak”* (Close contact identified later in 3^rd^ outbreak, 50, female, Da Nang)

As part of the public health strategy, an emphasis was placed on being ‘responsible’ citizens to protect national security, which encouraged people to limit their movements, even when not subject to the mandatory orders of local lockdowns. This fuelled and justified a mood of surveillance and suspicion of travel, which was initially directed towards those coming from outside of Vietnam and then, after the international borders closed, focused on those within Vietnam. As one community member participant recalls:

*“If any of my neighbours returned to our community without having their medical information and travel history declared, people in my area, including me, would criticize them and report them to the health authority … Admittedly sometimes we overreacted. Once an ambulance came to a household in our neighbourhood, the rumour spread quickly that an overseas Vietnamese had returned without declaring their health information and fell ill. We thought that person had hidden from the authorities and got really angry. But over the next couple of days it turned out to be wrong.”* (Community member, female, 42, Ha Noi)

Reflecting wider social views, these mischaracterisations tended to coalesce around existing tensions and served to amplify social prejudices against a specific group. Intense hostility was directed at those whose movements between locations were presumed to be motivated by pleasure and enabled by wealth and higher socioeconomic status.

*"Online newspapers reported on my husband's travel history, but the headline only focused on his visit to a massage salon. So people started to spread rumours. The content of the article was disparaging [suggesting he had committed adultery] and drove public opinion with negative emotions. But they didn't know my husband often had massages as therapy."* (Close contact identified early in 1^st^ outbreak, female, 41, Ho Chi Min)

#### 4.3.3 Fuel of social media amplified social blame

The wide reach of the media, both traditional and social media, played an important role in Vietnam's COVID-19 response by promoting public health messaging and facilitating contact tracing activities. However, the media were not bound to present this information ‘neutrally’ and the public health authorities had limited control over how their messages were used to garner attention on many social media sites as 'clickbait', breaking sensationalised stories in which individuals were misrepresented.

*"The media reported on the internet that my husband and I had travelled to visit our daughter outside the town, which was how we got infected. This spread fast through the villagers. But I stayed at home the whole time. How could I find time to go out like that given that there is an outbreak? But people blamed me: 'how dare you travel like that!’"* (COVID-19 survivor identified early in 2^nd^ outbreak, female, 58, Ha Noi)

For many participants, the accusations directed at them within formal media channels were then exaggerated and magnified through a frenzy of intensive attention on social media sites, generating a seemingly ‘indelible’ mark on their identities. Given the powerful prominence of social media in constructing, reforming and in some cases ruining identities, participants described how the ‘spoiling’ of their character online seeped back from the virtual to the physical world.

#### 4.3.4 Acquisition as an environmental risk: universalism diffuses blame

In contrast, the exposure and transmission routes of individuals identified later in the second and third waveswere framed not as an indicator of volition, but rather as being incidental to living within a particular community outbreak. This reflected a shifting understanding in which the acquisition of COVID-19 became framed as environmental rather than behavioural, with everyone vulnerable to infection. As cases started occurring at scale, signalling an intensification of an outbreak with cases distributed in large numbers across communities, the imagined linearity of the infection chain was disrupted. The presumption that infection was indicative of transgressive behaviour mollified over time and became less relevant as a sense-making strategy. Acquiring COVID-19 became depicted as unfortunate, with the risk disconnected from their personal actions:

*"No one saidanything about me. Because here, there were too many cases. How do we know who is to blame? During the outbreak, it was unknown who was first infected. People who were infected later did not know, so no stigma. In Da Nang, when the news broke, I did not even know where to trace [from whom I contracted the virus]."* (COVID-19 survivor identified later in 3^rd^ outbreak, male, 39, Da Nang)

Although evolving scientific knowledge of COVID-19 transmission virus may have played a role in this attitudinal change, it appeared to also be informed by it becomingincreasingly common for the majority to recognise that they l were at-risk themselves, once the virus was circulating within their own community. Community members interviewed articulated their realisation that as they themselves had become infected despite taking all the precautious measures, then the same may have applied to others before them too:

*"It was inevitable in the beginning for some cases and close contacts in my commune to receive some angry comments and rumours on the radio and newspaper because the entire community was in shock. I was scared too of the two cases in my area [during the second wave]. When cases rose to over 100, we started to understand that none of them want*ed *the disease, the sick themselves do not want to get infected.”* (Community member, male, 36, Ha Noi)

As assumptions made about intent evaporated, for some participants this provoked a revised assessment of the socially transgressive behaviour of previous cases. A few reflected on their previous reactions with some guilt and remorse:

*“My neighbours texted us on the Zalo group chat ‘Hey, Ms [name] did not have any symptoms, but she was positive with the coronavirus’ … But actually, we don’t think she knows that she had it, so we don’t blame her at all. So, we think that all the things that happened to her and us were just so unfortunate."* (Close contact identified later in 2^nd^ outbreak, female, 31, Ha Noi)

## 5 Discussion

We hypothesise that there is an inverse relationship between the number of cases and the 'stigma load' experienced by individuals associated with COVID-19, highlighting that epidemiological events, such as the lifting of lockdowns (a decision often based on low case numbers) can be seen as critical moments in shaping stigma through risk communication (Hargreaves et al., 2020). The fluidity of stigma indicates that the basis for stigmatising attitudes can be mitigated and even radically recalibrated across epidemiological and social contexts. Deliberate, preventive action can be taken to reduce the ‘stigma load’ experienced by individuals. The changing nature of stigma highlights the pertinent influence of how public health policies are interpreted and the socially ‘productive’ meanings projected onto them, which are framed by local moral contexts.

The case of COVID-19 stigma in Vietnam demonstrates again that policy-making during the COVID-19 has been biomedicine-centric while insights from non-biomedical disciplines are marginalised (Lohse & Bschir, 2020; Pickersgill et al., 2022). We have shown how problems which arise become difficult to address when the social consequences of policies are only an after-thought.

The misguided logic of linear infection, which underpinnedlay understandings of contract tracing, was used to justify blaming identified individuals for having brought the virus into communities. Although the public health strategy of contact tracing is not novel, the notion of ‘tracing’, i.e. presuming to discover the linear chains of transmission, abruptly exploded into the public consciousness. Without a more nuanced epidemiological understanding of index and primary cases, nor appreciating the high chance of asymptomatic spread, these epidemiological terms, which formed part of the public health strategy at the time, were endowed with powerful social meanings that served to justify the segregation of individuals within communities into the 'threatened' and the 'threat' (Roelen et al., 2020; Sotgiu & Dobler, 2020). This illustrates the pertinence of the early warnings from the World Health Organization and other experts about the negative ramifications of using stigmatising language to refer to people affected by the virus, such as ‘super spreaders’ (Logie & Turan, 2020). This is again a dominant concern with the language and misunderstanding of ‘monkey pox’ as a sexually transmitted infection given that in the early months of the outbreak it primarily affected men who have sex with men (Lane & Fauci, 2022; The Lancet Regional Health – Europe, 2022).

Public health strategies within the early stages of local outbreaks in Vietnam in 2020, coupled with the Government's emphasis on national security through surveillance by and between citizens (Pham, 2021; Tran et al., 2020) unintentionally legitimized seeking to identify affected individuals and provoked a socially evaluative commentary. However, while this collective effort can be interpreted as embodying community engagement (Ivic, 2020), our findings illuminate how this can concurrently and inadvertently create the conditions for stigma to flourish. Its inherent fluidity enabled these negative effects to be moderated once the public health risk was framed as environmental, rather than behavioural and individualised. The social response which formed in the later stages of each outbreak gives greater credence to the claims of social solidarity; albeit one that is diluted by the preceding social vilification that segregated individuals.

The manifestation and operation of stigma throughout the pandemic will inevitably continue to change, especially in the context of accessible vaccinations. However, understanding the dynamic life of stigma that is created, shaped, and modified by policy bears several lessons for current and future epidemics. In the context of COVID-19, our findings contribute to the argument that less targeted but more universalistic public health policies are necessary, as many scholars have suggested (Roelen et al., 2020). But in instances where policymakers are compelled to implement targeted strategies, specifically, contact tracing and public health messaging in this case, it will always be necessary to consider how these strategies can incorporate *a socially aware (S)* framing. This must be given due consideration in pandemic preparedness, especially to be able to pre-empt the trend to look for someone to blame that can readily occur in the socially febrile time of a public health emergency.

We suggest that it is essential for public health policies to be developed and implemented with *anticipation (A)* of their own consequence, and responsive *flexibility (F)* to the potential contributing effect they may have on social sense-making, rather than assuming their ‘neutrality’ in local social contexts, to actively minimise the risk of being appropriated as an instrument to justify the assignment of blame and to promote *equity (E)*. The risks in failing to do so and strategies for effectively managing social framings should be incorporated as a core component of public health training. Ensuring that public health strategies attend to the social effects (and after-life*)* of policies as an essential and integral consideration, from the inception of a public health policy plan, will protect and foster societal trust which is likely to sustain overall *resilience (R)* and engagement as we face the prospect of the increasing frequency of novel pandemics in the future. We propose that this learning can be encapsulated in the SAFER principles (Table 3), which have emerged from the findings of this case study.

**Table 3:** Guiding principles to invest in and protect health equity and social justice.

SAFER	
**S**ocially Aware	Consideration of ‘social life’ of interventions.
**A**nticipatory	Anticipate and plan for how to manage the social meanings and consequences of public health strategies in infectious disease outbreaks.
**F**lexible	Tailor to specific community needs. Adapt to emerging opportunities and threats. Responsive to relative risk (prioritises infection control but not neglect gravity of other concerns).
**E**quity	Promote equity by attending to inclusivity (language, social framing and interventions). Support fairer access, engage with the disenfranchised, and invest in local networks of community trust and knowledge.
**R**esilience	Fostering social justice, inclusivity and solidarity will support trust and resilient communities to engage in further public health responses.

## 6 Limitations and areas for further research

In collecting the data for this study, there were no practical means to safely interview participants in-person. The necessary reliance on remote data collection though provoked some problems in trust-building, which were managed by assuring confidentiality and establishing informal rapport. Remote interviewing rely on participants having the technological access and confidence to engage. This may have excluded some groups who require more targeted and tailored approaches to recuitment (Melis et al., 2022), which may have narrowed our sample. However, our recruitment strategy did allow us to interview survivors and former close contacts from groups disproportionately affected by infections. The inclusion of community members within our sample enabled us to broaden our inclusion criteria, with some older people successfully recruited. The community members’ sample enabled us to gather valuable insights into a highly sensitive topic, including the perspectives of those who recognised that their own responses had been stigmatising. Although we conducted targeted recruitment to increase the number of male participants within the survivor and former close contact category, we encountered a very high refusal rate among eligible men. We have provided some analytical interpretation of this finding, including proposing a gendered influence on the experience of COVID-19 stigma. Attending to intersectional influences which shape the manifestation and consequences of stigma in novel infectious disease outbreaks warrants further attention.

## 7 Conclusion

By exploring the experiences of affected individuals within the changing policy context of the first year of the COVID-19 epidemic in Vietnam, our study characterises variation in their experience of stigma (‘stigma load’). While our findings demonstrate how public health policies may unintentionally create a social stigma for specific groups, the fluidity of stigma captured in participants' experiences suggests possibilities to modify and mitigate it through attending to the social framing of epidemiological policies and attending to the SAFER principles.

## Funding statement

This study is also part of the “Understanding COVID-19 in Vietnam” programme and was funded by a grant from the 10.13039/501100000996Australian Department of Foreign Affairs and Trade (DFAT) Center for Health Security.

## Author contributions

DHT, SM, GJF, TAN, and SB conceptualised and designed the study. DHT and AN collected the data. DHT, SM, AN, TAN and SB were involved in data analysis and interpretation. DHT and SB prepared the initial draft of the paper, which was reviewed, edited and approved by all authors.

## Ethics

Ethics approval was obtained in both Vietnam (Ministry of Health – National Hospital for Tropical Diseases 10/HDDD-NDTU) and Australia (University of Sydney 2020/354). All participants were read the participants information sheets and provided verbal or written consent. Participants received 100,000 Vietnamese dong (approximately US$4.30) in phone credit as compensation for their time.

## Declaration of competing interest

The authors have no conflicts of interest.

## Data Availability

Data will be made available on request.
